# Clonidine Versus Chloral Hydrate for Recording Sleep EEG in Children

**Published:** 2020

**Authors:** Mahmoud Reza ASHRAFI, Hossein MOHEBBI, Mahmoud MOHAMADI, Elham AZIZI, Gholam Reza ZAMANI, Alireza TAVASOLI, Reza Shervin BADV, Firozeh HOSSEINI

**Affiliations:** 1 Department of Pediatrics , Children's Medical Center ,Pediatrics Center of Excellence , Division of Pediatric Neurology , Growth and Development Research Center ,Tehran University of Medical Sciences, Tehran, Iran; 2 Department of Pediatric Neurology, AJA University of Medical Sciences, Tehran, Iran; 3 Department of Pediatric Neurology, Besat Hospital, Hamadan University of Medical Sciences, Hamadan, Iran

**Keywords:** Premedication, Chloral Hydrate, Clonidine, Electroencephalography, Sedation, Sleep

## Abstract

**Objective:**

One of the difficulties to conduct electroencephalography (EEG) in pediatric patient population is that they are not always cooperative during the procedure. Different medications are used to induce sedation during EEG recording. In order to find a medication with the least adverse effects and high efficacy, the current study aimed at comparing clonidine and chloral hydrate as a premedication prior to EEG recording in pediatric population.

**Materials & Methods:**

A prospective, randomized, single-blinded, controlled trial was conducted on 198 children (9 to 156 months old) to investigate the sedative and adverse effects of clonidine and chloral hydrate. Patients, partially sleep-deprived the night before, were randomly divided into two groups of clonidine (n=100) and chloral hydrate (n=98) on an alternative day basis.

**Results:**

The average sleep onset latency was significantly longer in the clonidine group than chloral hydrate group (the Mann-Whitney test, p <0.0001). Sleep duration ranged 15 to 150 minutes and it was not significantly different between the two groups (the Mann-Whitney test, p = 0.2). Drowsiness terminated faster with chloral hydrate than clonidine.

Drowsiness after arousal was observed in 58% and 26.1% of patients in the clonidine and chloral hydrate groups, respectively; the difference between the groups was signiﬁcant (the Mann-Whitney test, p = 0.058). EEG results were reported normal in 77 subjects in the chloral hydrate group (77%) and 69 subjects (69%) in the clonidine group (p = 0.161). Generalized epileptiform discharges were significant in the clonidine group (the Mann-Whitney test, p = 0.006).

**Conclusion:**

The results of the current study showed that both 5% chloral hydrate (1 mL/kg) and clonidine (4 μg/kg) could be administered as a premedication prior to EEG recording in children, although drowsiness after arousal was higher with clonidine than chloral hydrate. However, the yield of generalized epileptiform discharges in the clonidine group was greater than that of the chloral hydrate group.

## Introduction

One of the difficulties to conduct electroencephalography (EEG) in pediatric population is that they are not always cooperative during the procedure. It is generally agreed that most children undergoing medical procedures get frightened and uncooperative, and should be managed with behavioral and non-pharmacologic techniques. Unfortunately, a small percentage of pediatric patients cannot be successfully managed solely with such techniques (1). Several sedative-hypnotic agents can be used in children for medical procedures including benzodiazepines, ketamine, hydroxyzine, melatonin, clonidine, chloral hydrate, sufentanil, dexmedetomidine, etc. 

Clonidine, an α-2 agonist, is suggested as an alternative agent for sleep induction in children. Mikawa et al. (1993), (2) concluded in a review study that clonidine, administered via oral, rectal, or caudal route is a promising adjunct to anesthetics and analgesics to enhance the quality of perioperative care in infants and children. Later publications also support the utilization of clonidine as premedication (3).

Chloral hydrate has some known adverse effects such as nausea, vomiting, agitation, ataxia, prolonged sedation, delayed apnea events, gastric irritation, potential carcinogenicity, and genotoxicity even as a single low-dose (4–6). 

A number of previous studies compared sedative and adverse effects of some premedication such as midazolam, melatonin and chloral hydrate, but there is still a lack of studies on comparing clonidine with chloral hydrate. The current prospective, randomized, single-blinded, controlled trial aimed at comparing the sedative and adverse effects of clonidine and chloral hydrate.

## Methods & Materials

The protocol of the current prospective study was approved by the Ethics Committee of Tehran University of Medical Sciences. Written and oral informed consent was obtained from parents prior to inclusion in the study. The current study was conducted over one year at a major pediatric university hospital in Tehran, Iran. The enrolled subjects were children within the age range of 9 to 156 months scheduled for EEG recording in the center. The study had a randomized, single-blinded design; patients were randomly allocated to one of the two premedication options of oral clonidine or chloral hydrate.

The following data were obtained: age, gender, weight, EEG indication, underlying medical conditions, and medication history. Information concerning clonidine and chloral hydrate included the dose, sleep onset time, sleep duration, time to arousal, efficacy, side effects, and complications. A trained staff filled out a questionnaire for each patient to collect the following data: neurological diagnosis, sleep onset latency, sleep duration, drowsiness time, and adverse events that occurred within the first four hours after EEG recording. 

The administered doses of clonidine and 5% chloral hydrate were 4 μg/kg and 1 mL/kg, respectively. Premedication was administered 30 minutes prior to EEG recording. An analog 21-channel Nihon Kohden EEG machine based on the standard international 1020 system was used. Two trained and skilled pediatric neurologists interpreted recorded EEGs.

 Patients who were thermodynamically unstable or had severe respiratory failure, patients with severe failure to thrive, infants younger than six months of age, the ones with a history of hypersensitivity to either clonidine or chloral hydrate, and those that their parents refused to provide consent were excluded. 

**Statistical Analysis:**

Based on the literature, the required study sample size was 100 in each group (α= 0.05, β= 0.2). Data were analyzed for normal distribution using the Shapiro-Wilk test. Statistical analysis was performed using independent samples t-test and the Mann-Whitney-Wiloxon test for continuous data. Categorical variables were analyzed using the Fisher exact and Chi-squared tests. A P-value of <0.05 was considered significant. A biostatistician blinded to the study groups performed the statistical analysis.

## Results

Totally, 198 pediatric patients were enrolled in the study; 81 females (40.7%) and 117 males (58.8%). The patients’ age ranged 9 to 156 months (mean ± standard deviation (SD): 42.11 ±42.64 months). The two groups were matcher for gender. The clonidine group consisted of 58 (49.6%) males and 42 (51.9%) females and the chloral hydrate group consisted of 59 (50.4%) males and 39 (48.1%) females (p = 0.75). The mean age of the subjects in the clonidine group was higher than that of the ones in the chloral hydrate group (57.430.5 vs. 27 21.8 months); the difference between the groups was significant (p >0.0005). 

**EEG indications:**

Top indication for referral for EEG in both groups was seizure disorders (84 (54.9%) subjects in the chloral hydrate and 76 (45.1%) patients in the clonidine groups); however, and the frequency of this etiology was not significantly different between the groups (p=0.116). Other indications were attention deficit hyperactivity disorder (ADHD) (2.5%), sleep disorders (0.5%), speech disorders (0.5%), and miscellaneous (11%). ADHD, as an indication for EEG, was observed only in the clonidine group (12 children vs. zero children) (p <0.001), but other etiologies were evenly distributed amongst subjects of both groups (p=0.05).

**Efficacy of premedication:**

The time gap between premedication and the start of sleep (sleep onset latency) varied from 9 to 190 minutes in the studied subjects. The average sleep onset latency in the clonidine group was significantly longer than that of the chloral hydrate group (86 minutes and 17 seconds vs. 48 minutes and 48 seconds) (p <0.0001).

Sleep duration in total subjects ranged 15 to 150 minutes and there was no significant difference between the two medications (74 minutes and 12 seconds for clonidine vs. 68 minutes and 8 seconds for chloral hydrate) (p = 0.2). 

**Figure 1:**
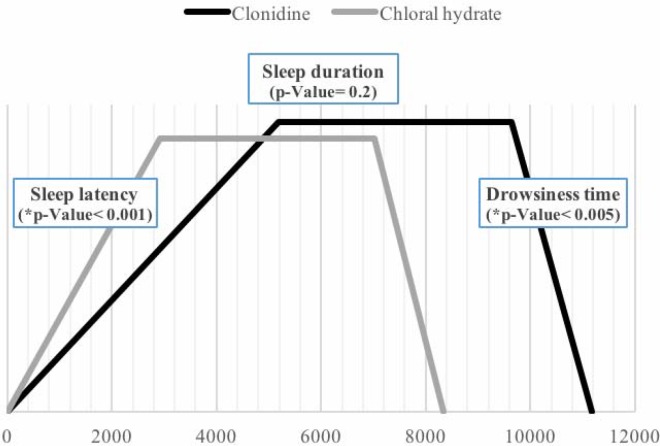
Sleep Characteristics in the Chloral Hydrate and Clonidine Groups

For patients medicated with clonidine, it took longer to be wide awake (drowsiness time: 26 minutes and 13 seconds for the chloral hydrate group vs. 32 minutes and 21 seconds for the clonidine group) (p <0.0001). Sleep characteristics of the two groups are provided in Figure 1.

**Premedication adverse effects:**

Unusual drowsiness occurred in 58 (58%) patients medicated with clonidine, while it was observed in 26 (26.1%) patients in the chloral hydrate group (p = 0.058). The only other adverse effect was vertigo, occurred in only 3% of patients medicated with clonidine. Other side effects such as nausea, vomiting, or agitation were not observed.

**EEG interpretation:**

Recorded EEGs were twice interpreted by two trained and skilled pediatric neurologists. The EEG interpretations were similar in 70% of the cases. EEG results were reported normal in 77 subjects (77%) in the chloral hydrate group and 69 subjects (69%) in the clonidine group (p = 0.161). Focal epileptiform discharge and multifocal epileptiform discharge were not significantly different between the two groups (p = 0.74 and 0.2, respectively). The reports on the focal background disturbance, diffuse background disturbance, and fast activity in the two groups were not significantly different (p = 0.1, 0.15, and 0.15, respectively). Generalized epileptiform discharge was reported in 14 subjects in the clonidine group and three subjects in the chloral group (p = 0.006).

## Discussion:

Children undergoing EEG may experience significant anxiety and distress during the procedure, but the question whether routine premedication for children prior to EEG is necessary or not is currently under debate. Hatava and Olsson showed that pre-operative psychological preparation and the presence of parents were beneficial and could reduce the need for pharmacological premedication (7). However, sedative premedication with midazolam was more effective than either parental presence or no intervention at all to manage child/parent anxiety during the pre-operative period (8,9). According to these facts, researchers are looking for the best medication to induce sleep during EEG in children. An ideal medication for this goal should have rapid onset, long-acting sedative effect, low side effects, and the minimum impact on the EEG background (8). Benzodiazepines are drugs most widely used as premedication in pediatric anesthesia, and midazolam is at the top of the list of benzodiazepines used for induction of sedation in children (10). Midazolam, when used as the premedication in children, has a number of beneficial effects such as effective sedation, rapid onset, short acting, lower rate of vomiting, and less amnesia after sedation (11–13); although some paradoxical reactions such as agitation (14,15) and hypotonia (8) are observed following the utilization of midazolam in children.

Some studies were also performed to compare chloral hydrate and midazolam (16), chloral hydrate, and melatonin (17) as premedication in children. 

 Recently, clonidine is suggested by anesthesiologists for the induction of sedation before anesthesia. A study investigated the application of clonidine as a pre-anesthetic agent in the children undergoing surgery and indicated that oral clonidine was an effective premedication with few side effects (16). However, the current study suggests that further studies should be conducted to determine the optimal dosage and the safety of this drug in children. To the best of authors` knowledge, no studies compared clonidine and chloral hydrate for pediatric premedication, especially in children undergoing EEG. 

This study designed for the clinical advantages and disadvantages of oral clonidine in comparison with chloral hydrate as premedication prior to EEG recording in children. 

Several studies on premedication in the pediatric population showed that clonidine had slow onset of action (16,17,18). The current study finding on the clonidine onset of action was in agreement with those of the previous studies. Chloral hydrate induced sleep in the current study subjects faster than clonidine, although the sleep durations were similar in the two groups. The subjects medicated with clonidine had longer drowsiness period on average after the procedure compared to the ones medicated with chloral hydrate. The time of maintaining sedation with clonidine is long enough, which makes this drug a more suitable choice for premedication prior to EEG recording in pediatric patients. Other adverse effects such as nausea, vomiting, or seizure were not observed in any of the groups. In the current study, no agitation or hypotonia was reported, similar to what was observed with midazolam previously (16).

In the current study, there was no significant difference in gender distribution between the two groups, although the mean age of the subjects in the clonidine group was higher.

One important factor as premedication agent in EEG recording is its impact on EEG interpretation. According to EEG results, normal EEG was observed in 77% of the subjects in the chloral hydrate group versus 69% in the clonidine group, which the difference between the groups was not significant (p = 0.16). Other EEG findings such as focal epileptiform discharge, multifocal epileptiform discharge, focal background disturbance, multifocal background disturbance, generalized background disturbance, and rapid activity were not significantly different between the two groups. The only exception was generalized epileptiform discharge, which was significantly higher among the clonidine-medicated subjects and this meant a higher diagnostic yield of clonidine group. Clonidine also had no impacts on the background of EEG, unlike midazolam with rapid EEG activity (16) and melatonin with generalized rapid beta activity followed by slow delta activity in temporal regions (17).

There were no significant differences in the indications of EEG between the two groups except for patients with ADHD, which the EEG technicians tended to administer clonidine to such cases.

Chloral hydrate induced sleep faster than clonidine, but the sleep duration was similar for subjects in the two groups. The subjects medicated with clonidine had longer drowsiness time on average compared to the ones medicated with chloral hydrate.

Longer drowsiness time in clonidine group was the only difference between drug adverse effects in two groups .Other drug adverse effects such as nausea, vomiting, agitation, or seizure were not observed in both groups. Agitation and hypotonia that was reported in previous midazolam study was not seen in this study. (16). Additionally, no respiratory failure or hypoxia was observed using these drugs, although infants younger than six months of age were excluded from the study.

**In Conclusion,** Results of the current study showed that both 5% chloral hydrate (1 mL/kg) and clonidine (4 μg/kg) can be administered as a sedative agent prior to EEG recording in children. Clonidine with a high efficacy in inducing and maintaining sleep during EEG recording and minimal adverse effects can be one of the ideal choices for premedication prior to EEG recording in pediatric patients. The yield of generalized epileptiform discharges in the clonidine group was greater than that of the chloral hydrate group, with little confusing effects on EEG interpretation. 
